# Rapid high resolution melting assay to differentiate *Streptococcus suis* serotypes 2, 1/2, 1, and 14

**DOI:** 10.1002/mbo3.995

**Published:** 2020-01-22

**Authors:** Simone Scherrer, Fenja Rademacher, Nathalie Spoerry Serrano, Jacques Schrenzel, Marcelo Gottschalk, Roger Stephan, Patricia Landolt

**Affiliations:** ^1^ Department of Veterinary Bacteriology Institute for Food Safety and Hygiene Vetsuisse Faculty University of Zurich Zurich Switzerland; ^2^ Bacteriology Laboratory Geneva University Hospitals and University of Geneva Geneva Switzerland; ^3^ Swine and Poultry Infectious Diseases Research Center (CRIPA) Groupe de recherche sur les maladies infectieuses des animaux de production Faculty of Veterinary Medicine University of Montreal Saint‐Hyacinthe QC Canada

**Keywords:** differentiation serotypes 2, 1/2, 1 and 14, high resolution melting, Streptococcus suis

## Abstract

This rapid high resolution melting (HRM) assay allows distinguishing between *Streptococcus suis* serotype pairs 2 and 1/2 as well as 1 and 14, respectively, based on a single‐nucleotide polymorphism within capsular polysaccharide synthesis gene cluster K. This assay is easy to implement and identifies potential zoonotic serotypes.

## Graphical abstract

The designed high resolution melting assay allows to correctly differentiate *Streptococcus suis* serotype pair 2 and 1/2 or 1 and 14, respectively. The assay is based on a single‐nucleotide polymorphism within the capsular polysaccharide synthesis gene cluster K and enables a rapid identification of potential zoonotic serotypes.

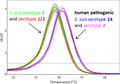

## 1 INTRODUCTION

*Streptococcus suis* (*S. suis*) is an important pathogen of pigs and considered to be responsible for various diseases such as septicemia with sudden death, meningitis, endocarditits, and arthritis (Gottschalk, Segura, & Xu, 2007). Currently, there are at least 29 *S. suis* serotypes (Okura et al., 2016) described based on a serological reaction against the capsular polysaccharide (CPS), which has been described to be a major virulence factor with antiphagocytic properties (Baums & Valentin‐Weigand, 2009). *S. suis* serotype 14 and in particular heterogenous serotype 2 are emerging zoonotic pathogens often associated with disease in both pigs and humans worldwide (Goyette‐Desjardins, Auger, Xu, Segura, & Gottschalk, 2014). Formerly, serological typing was performed with different antisera of known type, based on three different techniques: Neufeld's capsular reaction, capillary precipitation, and coagglutination tests (Gottschalk, Higgins, Jacques, & Dubreuil, 1992). In recent times, more frequently multiplex PCR assays are used targeting genes based on CPS (Okura et al., 2014), which are identical for serotype pairs 2 and 1/2, and 1 and 14 except for a single base pair substitution in codon 161 of *cpsK* gene (Athey et al., 2016). A challenge for diagnostic laboratories is the fact that available PCR tests do not allow resolving these aforementioned serotype pairs 2 and 1/2, and 1 and 14.

Recently, an in silico pipeline using whole‐genome sequencing (WGS) short‐read data was developed, which is able to differentiate these serotypes (Athey et al., 2016). Nevertheless, for a rapid identification of potential zoonotic strains with serotypes 2 and 14, a reliable inexpensive and feasible high‐throughput approach for routine diagnostic laboratories would be a valuable tool. The aim of this study was to evaluate the potential of *cpsK* as target for differentiating *S. suis* serotypes 2, 1/2, 1, and 14 from pure culture using a novel high resolution melting (HRM) assay.

## 2 MATERIALS AND METHODS

For this purpose, four reference strains comprising serotypes 2, 1/2, 1, and 14, one human, and 12 porcine *S. suis* isolates of serotypes 1 or 14 and 2 or 1/2 were used to develop a novel HRM assay (Table 1). Moreover, 58 *S. suis* isolates of other serotypes, as well as four further *Streptococcus* spp*.* isolates were included in the study. All strains were grown on Columbia agar with sheep blood (Thermo Fisher Diagnostics AG) and incubated at 37°C for 48 hr under aerobic conditions. Strains were identified by matrix‐assisted laser desorption ionization time‐of‐flight mass spectrometry (MALDI‐TOF MS, Bruker). Genomic DNA was extracted using a standard heat lysis protocol (Sambrook & Russel, 2006).

**Table 1:** Bacterial strains used for HRM development

Strains	Strain designation	Source	Year of isolation	Serotype^a^	Definitive Serotype^b^	Sequence type^c^ (ST)
*S. suis*	ZH 468	Mitral valve/pig^d^	2007	2 or 1/2	1/2	ST28
*S. suis*	ZH 1192	Lung/pig^d^	2015	2 or 1/2	1/2	ST28
*S. suis*	ZH 423	Heart/pig^d^	2016	2 or 1/2	1/2	ST1133
*S. suis*	ZH 1329	Brain/pig^d^	2015	2 or 1/2	2	ST1103
*S. suis*	ZH 462	Heart/pig^d^	2016	2 or 1/2	2	ST28
*S. suis*	ZH 269	Heart/pig^d^	2015	1 or 14	1	ST13
*S. suis*	ZH 1598	Brain/pig^d^	2016	1 or 14	1	ST13
*S. suis*	ZH 1635	Joint/pig^d^	2017	1 or 14	1	ST13
*S. suis*	ZH 1656	Joint/pig^d^	2017	1 or 14	1	ST13
*S. suis*	ZH 730	Joint/pig^d^	2018	1 or 14	1	ST13
*S. suis*	ZH 735	Joint/pig^d^	2018	1 or 14	1	ST13
*S. suis*	ZH 731	Heart/pig^d^	2018	1 or 14	1	ST13
*S. suis*	*S. suis*14	Blood/human^e^	2018	1 or 14	14	ST1
*S. suis*	Ref. Serotype 1	Blood/pig^f^		1	1	ST13
*S. suis*	Ref. Serotype 2	Brain/pig^f^		2	2	ST1
*S. suis*	Ref. Serotype 1/2	Tonsil/pig^f^		1/2	1/2	ST56
*S. suis*	Ref. Serotype 14	Not known/human^f^		14	14	ST6
58 *S. suis*	—	Various tissues/pig^d^	2007–2017	13 different serotypes^g^	—	29 different ST
4 *S.* spp.	—	Various tissues/pig^d^	2007–2012	—	—	—

a Serotype characterization by multiplex PCR (Kerdsin et al., 2014).

b Definitive serotype assignment after *S. suis* HRM.

c Sequence type characterization by multilocus sequence typing (King et al., 2002).

d Strains isolated between 2007 and 2018 from the Department of Veterinary Bacteriology, University of Zurich, Switzerland.

e Bacteriology Laboratory, Division of Laboratory Medicine, Geneva University Hospitals, Geneva, Switzerland.

f Reference strains from Swine and Poultry Infectious Diseases Research Center, Groupe de recherche sur les maladies infectieuses des animaux de production, University of Montreal, Saint‐Hyacinthe, Canada.

g Serotypes 3, 4, 5, 6, 7, 8, 9, 12, 15, 16, 21, 28, and 31 (belonging to 29 different sequence types) were tested.

**Figure 1:**
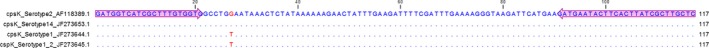
Representation of sequence alignment of the *cpsK* amplicon generated by the HRM‐PCR using primers cpsK_for and cpsK_rev (illustrated as pink arrows) of serotypes 2, 14, 1, and 1/2. Single‐nucleotide polymorphism (SNP) region is highlighted in red, whereas conserved nucleotides are shown in blue. The SNP indicated gives rise to different melting behavior in the HRM assay. Accession numbers of GenBank of corresponding sequences are indicated

The *cpsK* region flanking the nonsynonymous single‐nucleotide polymorphism (SNP) at base pair position 483 (Athey et al., 2016; Roy et al., 2017) (G for serotypes 2 and 14, T or C for serotypes 1 and 1/2) was targeted using the following primers: (cpsK_for: 5′‐ GATGGTCATCGCTTTGTGGTG‐3′) and (cpsK_rev: 5′‐ GAGCAAGCGATAAGTGAAGTATTCATC‐3′) and producing an amplicon of 117 bp (Figure 1). All qPCR experiments with subsequent HRM analysis were performed on a Rotor‐Gene Q (Qiagen, Hilden, Germany) using Type‐it HRM PCR Kit (Qiagen). The total reaction volume was 15 µl. About 1 µl of sample DNA of 100 pg was added to a reaction mixture containing 7.5 µl 2× Type‐it HRM Mastermix, 0.5 µM of each primer and ultrapure water. The PCR thermocycling conditions were as follows: initial denaturation at 95°C for 5 min, 40 cycles with denaturation at 95°C for 10 s, and annealing/extension at 66°C for 30 s followed by a second cycling step at 95°C for 10 s and 40°C for 2 min. Finally, a HRM ramping from 70°C to 82°C was performed. Fluorescence data were acquired at 0.1°C increments every 2 s to generate specific melting curves. Reference strains with serotypes 2, 1/2, 1, and 14 were included as melting curve standards and positive controls. To exclude contaminations in the reaction mixture, ultrapure water was used as a negative control in each experiment. Data analysis was performed using Rotor‐Gene Q Software 2.3.1 (Qiagen). Normalized and difference plots were generated. To normalize the results, the premelt and postmelt signals of all samples were set to uniform relative values from 100% to 0%. In order to generate difference plots, normalized fluorescence data of sample curves were subtracted from the curve of reference strain serotype 1/2 to visually accentuate differences in a greater resolution.

The HRM assay was evaluated, and its specificity was determined. To examine the intra‐ and interassay variability of the melting temperatures (*T*_m_), representing the repeatability of the developed HRM assay, all isolates were tested. The variability assays were performed in triplicates in three independent runs at three different days.

## 3 RESULTS

**Figure 2:**
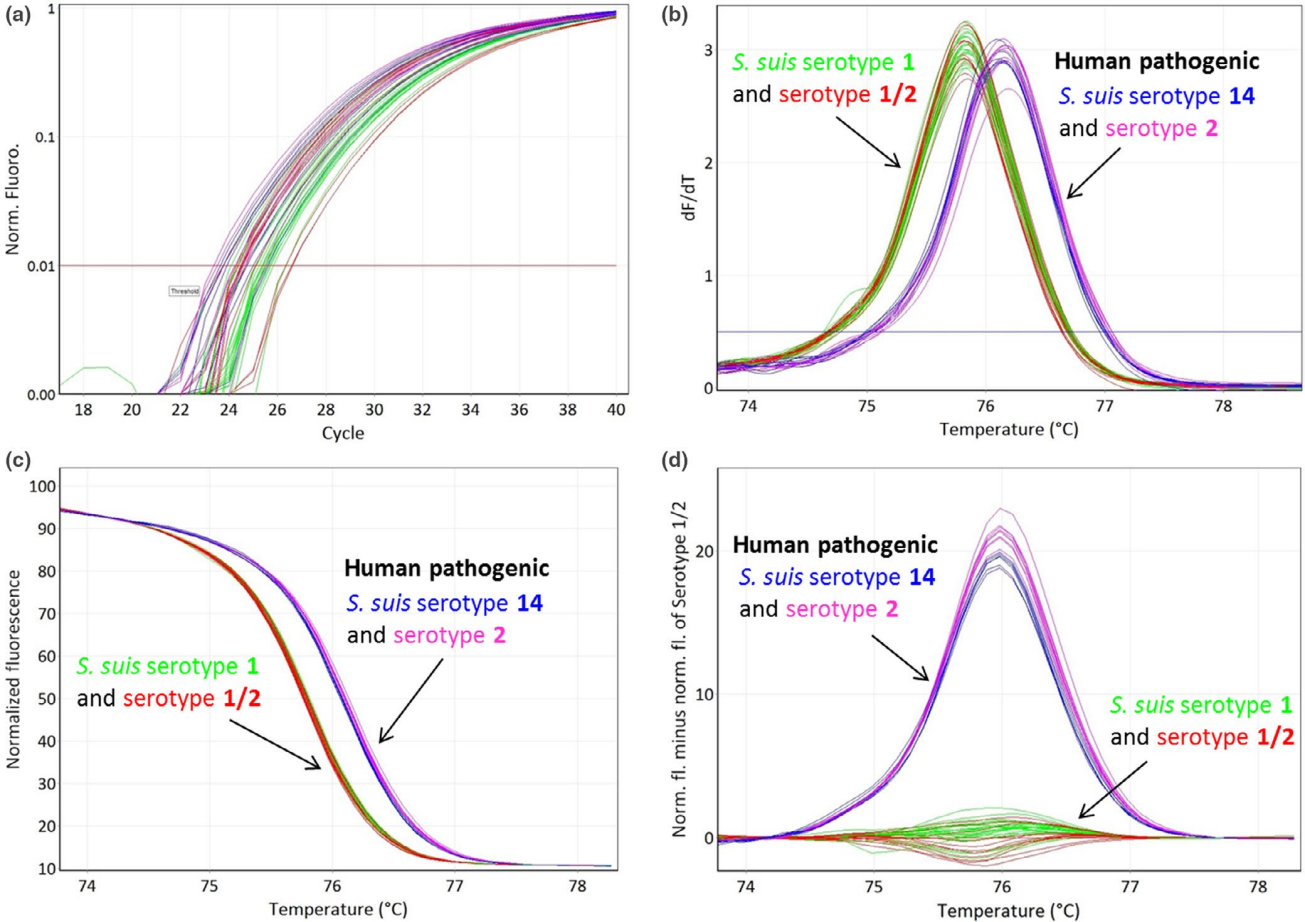
Representation of a high resolution melting (HRM) assay of 12 Swiss porcine *Streptococcus suis* strains, one human *S. suis* strain of serotype 14, and four reference strains with serotypes 14, 2, 1, and 1/2 obtained by triplicate‐testing of each strain for intra‐assay variability determination. The two groups of melting curves obtained allow rapid identification of human pathogenic *S. suis* serotypes (serotypes 2 and 14). Serotype 1/2 (red), serotype 14 (blue), serotype 1 (green), and serotype 2 (pink) are illustrated in each plot. (a) qPCR amplification plot; (b) melting curves of the HRM step; (c) normalized plot; (d) difference plot in relation to reference strain serotype 1/2

The HRM assay clearly divided the 17 *S. suis* strains into two clusters grouping serotype 2 with 14 and serotype 1/2 with 1 (Figure 2). By combining serotype determination obtained by PCR (Kerdsin et al., 2014) into serotype pairs 2 and 1/2 or serotype pairs 1 and 14 with the obtained results of the HRM assay, it is possible to separate and correctly assign the corresponding serotype to each *S. suis* isolate. Based on our results, the target was 100% specific for the four serotypes tested as none of the *Streptococcus* spp. or *S. suis* with different serotypes (Table 1) yielded a PCR positive result. The intra‐assay coefficients of variability (CVs) of *T*_m_ were between 0.008% and 0.03% and interassay CVs, respectively, between 0.04% and 0.07% illustrating a highly reproducible and stable assay. Serotypes 1 and 1/2 yielded a *T_m_* of 75.9 ± 0.1°C, whereas serotypes 2 and 14 yielded a *T*_m_ of 76.2 ± 0.1°C, respectively (Figure 1).

## 4 DISCUSSION

In this report, a novel HRM assay based on SNP detection in PCR products is described. HRM analysis is a rapid and low cost genotyping method simply convertible in laboratories as a singleplex method with a low risk of contamination (Reed, Kent, & Wittwer, 2007). The reliable distinction between serotypes 2, 14, 1, and 1/2 is useful for classification of circulating *S. suis* strains and helps strain tracking in case of disease outbreaks. In addition, this assay provides an easily applicable diagnostic tool for high‐throughput screening and rapid identification of *S. suis* serotypes 2 and 14 relevant for human infection without the need of a WGS approach. Compared with Sanger sequencing and WGS, a HRM assay is not depended on manual inspection of the sequencing data and results are obtained straightforward in less than 1.5 hr. Reagent costs are low with about 1 CHF per sample in comparison to Sanger sequencing, which was around ten times as expensive with an estimated prize of 10 CHF per sample. The cost of WGS sequencing of approximately 200 CHF per sample is not competitive if the focus of investigation is set at serotype differentiation or at screening of potentially zoonotic isolates. Even though, the local reagents and labor cost may be variable, HRM is by far the most cost efficient and easily applicable assay delivering accurate results in a short turnaround time. Thus, a possible surveillance system contributing to the development of proficient public health policies can be established.

## 5 CONCLUSION

To conclude, we have developed a specific HRM assay distinguishing between serotype pairs 2 and 1/2 and 1 and 14, respectively, based on a SNP within *cpsK*. This assay allows a rapid separation of these *S. suis* serotypes in routine diagnostic laboratories using molecular based serotyping in order to assess a zoonotic risk of emerging strains.

## CONFLICT OF INTERESTS

None declared.

## AUTHOR CONTRIBUTIONS

Simone Scherrer: Conceptualization‐Equal, Investigation‐Equal, Validation‐Lead, Writing‐original draft‐Lead; Fenja Rademacher: Investigation‐Supporting; Nathalie Spoerry Serrano: Investigation‐Supporting; Jacques Schrenzel: Resources‐Equal, Writing‐review and editing‐Supporting; Marcelo Gottschalk: Resources‐Equal, Writing‐review and editing‐Supporting; Roger Stephan: Conceptualization‐Equal, Supervision‐Lead, Writing‐review and editing‐Lead; Patricia Landolt: Conceptualization‐Equal, Investigation‐Equal, Validation‐Supporting, Writing‐review and editing‐Supporting.

## ETHICS STATEMENT

None required.

## Data Availability

Raw datasets from intra‐ and interassay variability runs are available upon request.
